# The Kansas Veterinary Medical Society

**Published:** 1890-07

**Authors:** S. L. Hunter

**Affiliations:** Secretary; Fort Leavenworth, Kansas


### SOCIETY PROCEEDINGS.

*The Kansas Veterinary Medical Society.*—The Kansas Veterinary Medical Society met at the Whitley House, in Emporia, March 13th, 1890. The meeting was called to order promptly at 7.30 P.M., with President George C. Pritchard in the chair. The minutes of the previous meeting were read and approved. The constitution and by-laws were presented, and after a few excellent changes, were accepted.

Although the Kansas Veterinary Medical Society is in its infancy, yet it has taken a long stride in the right direction, as no one can be elected to membership unless he holds a diploma from a recognized veterinary college or university with power to grant such diplomas. Certificates of membership will be issued to members properly filled out and signed by the president and secretary.

A most excellent essay was read by S. E. Phillips, V.S., of Wichita, on "A Rectal Examination as an Aid in the Diagnosis of Certain Diseases." S. L. Hunter, V.S. (U. S. Army), Fort Leavenworth, read an essay on "Veterinary Education." Both papers were well received and created an interesting discussion by D. Lemay, V.S. (U. S. Army), Fort Riley; W. H. Richards, V.S., Emporia; George C. Pritchard, V.S., Topeka, and J. M. Phillips, V.S., of Wichita.

The President related an interesting case of lead-poisoning, and Mr. Bird gave his experience, with others, in acute and chronic pleuro-pneumonia in New York. Dr. Lemay also gave his experience with the same disease while he was State Veterinarian of Maryland. Essayists for the annual meeting to be held in Topeka, in September, 1890, were appointed by the President as follows: D. Lemay, V.S., Don C. Ayer, V.S., J. M. Phillips, V.S., and S. C. Orr, V.S.

A vote of thanks was given the essayists. The motion to adjourn was then carried, and the members of the Society were taken by W. H. Richards, V.S., Emporia, and given a most excellent supper, which was heartily enjoyed by all, and before breaking up a vote of thanks was enthusiastically given Mr. Richards for the hospitable manner in which all members had been received and cared for by him, and with many regrets we left for home, all voting it the most instructive meeting we had ever had, which one can judge by the interest manifested throughout. The Kansas Veterinary Medical Society will take its place in the front ranks in promoting the interests of veterinary science at all times and upon all occasions. D. Lemay, V S., was chosen delegate from our Society to the United States Veterinary Medical Society, at Chicago, in September.

The Society is officered as follows: President, George C. Pritchard, V.S , Topeka; Vice-President, R. C. Moore, V.S., Holton; Treasurer, W.

H. Richards, V.S., Emporia; Secretary, S. L. Hunter, V.S., Fort Leavenworth; Censors, Don C. Ayer, V.S., Leavenworth; J. M. Phillips, V.S., Wichita; S. E. Phillips, Wichita.

S. L. HUNTER, V.S., *Secretary.*
*Fort Leavenworth, Kansas.*

